# Cholecystocolic Fistula: A Diagnostic Enigma

**DOI:** 10.4103/1319-3767.45054

**Published:** 2009-01

**Authors:** Mervyn F. S. Correia, Dilip P. Amonkar, Swati V. Nayak, Jean-Louis A. S. Menezes

**Affiliations:** Department of General Surgery, Goa Medical College, Bambolim, Goa, India

**Keywords:** Biliary-enteric fistula, cholecystectomy, cholecystocolic fistula ERCP

## Abstract

Cholecystocolic fistula is a rare biliary-enteric fistula with a variable clinical presentation. Despite modern diagnostic tools, a high degree of suspicion is required to diagnose it preoperatively. Biliary-enteric fistulae have been found in 0.9% of patients undergoing biliary tract surgery. The most common site of communication of the fistula is a cholecystoduodenal (70%), followed by cholecystocolic (10–20%), and the least common is the cholecystogastric fistula accounting for the remainder of cases. These fistulae are treated by open as well as laparoscopic surgery, with no difference in intraoperative and postoperative complications.

We report here a case of obstructive jaundice, which was investigated with a plain film of the abdomen, abdominal ultrasonography, and endoscopic retrograde cholangiopancreatography, but none of these gave us any clue to the presence of the fistula was discovered incidentally during an open surgery and was appropriately treated.

Spontaneous biliary-enteric fistulae have been rarely reported. In fact, most such fistulae result as complications of gall stone disease. A sequence of events occurs in acute calculous cholecystitis. During these attacks, the adjacent serosal surface becomes inflamed and adherent to the gall bladder. The ischemic area in the wall of the gall bladder becomes gangrenous, and because of the increased pressure within, its contents penetrate its own necrotic wall first, and then, the wall of the adjacent colon, forming a cholecystocolic fistula.[1]

Another cause is from pressure necrosis of an impacted gall stone usually at the neck of the gall bladder, which gradually erodes into the colon.

## CASE REPORT

**Figure 1:**
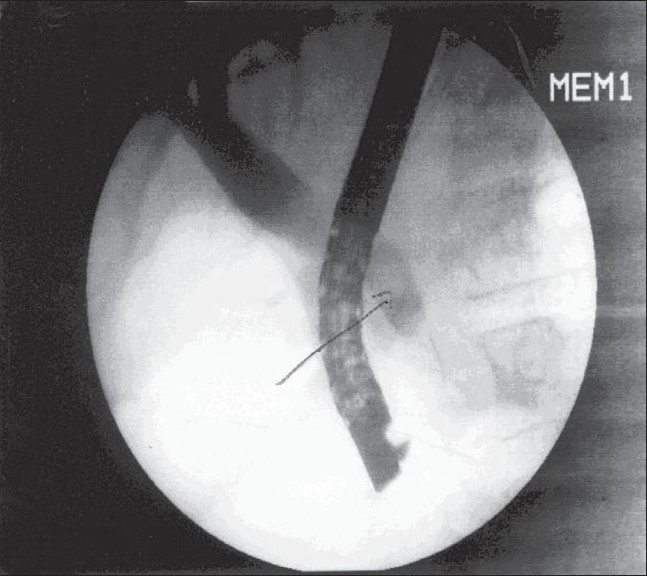
ERCP showing calculus in common bile duct

**Figure 2:**
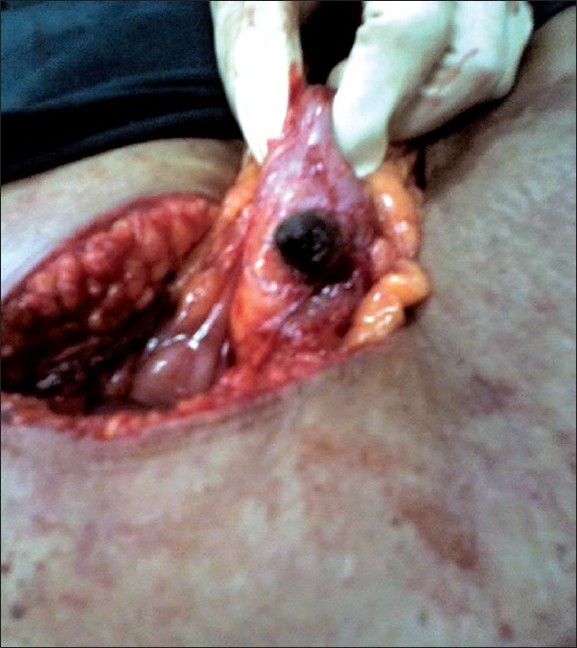
Right transverse colon with gall stone in fistula

A 59-year-old woman presented to the emergency department of our hospital with a history of fever, increasing jaundice, and right upper quadrant abdominal pain for two weeks. On examination, she was found to be febrile, deeply icteric, and tender in the right hypochondrium. Blood examination revealed a serum bilirubin level of 11.7 mg% (normal range, 0.7–1.1 mg%) and a serum alkaline phosphatase level of 320 IU (normal range, 40–125). An abdominal ultrasonography revealed an enlarged liver, dilated intrahepatic biliary radicals, and a dilated common bile duct with a 12× 10-mm calculus in the infraduodenal common bile duct (CBD) [Figure 1]. The gall bladder was thickened with multiple gall stones, the largest being 15 × 15 mm in the neck of the gall bladder [Figure 2]. No air was seen in the biliary tract.

As the patient showed features of cholangitis, she was given parenteral cefazolin and vitamin K. Forty-eight hours later, after the pyrexia had settled, an endoscopic retrograde cholangiopancreatography (ERCP) was done; the cholangiogram revealed a dilated CBD with a filling defect in the lower end of the CBD, which was suggestive of a calculus. The cholangiogram did not show any fistulous communication between the gall bladder and the colon. A sphincterotomy was done along with an extraction of the stone and clearance of the CBD with a temporary stenting of the common bile duct.

**Figure 3:**
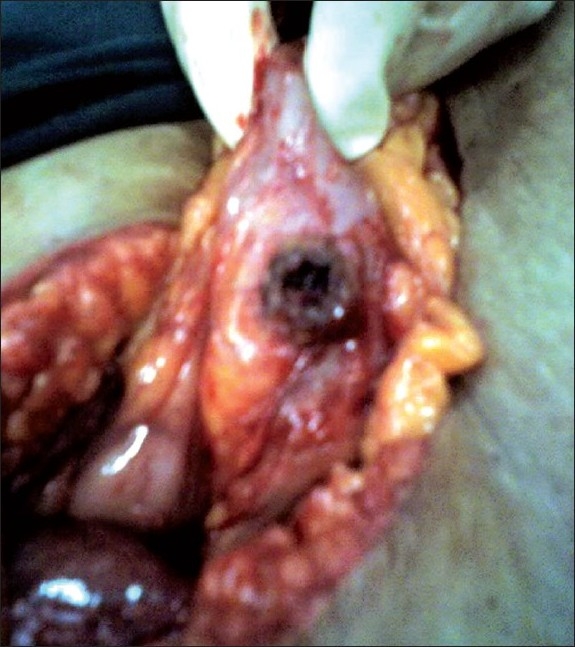
Fistula in the right transverse colon

Two weeks later, the patient was posted for laparoscopic cholecystectomy. During laparoscopy, it was evident that there were dense adhesions between the gall bladder, transverse colon, and the omentum. The Calot's triangle was virtually inaccessible. It was therefore decided to convert to an open cholecystectomy, and a right upper paramedian incision was used. Dissection in the Calot's triangle was extremely difficult although the stent in the CBD was palpable. Hence, it was decided to proceed by the “fundus first” method. Dense adhesions between the gall bladder and omentum were separated, and an attempt was made to create a plane between the neck of the gall bladder and the right colon at the site of the stone impaction which could be clearly felt. As this was impossible, the gall bladder neck was incised over the stone on the gall bladder to prevent damaging the right transverse colon, thereby keeping a cuff of the wall of the gall bladder on the colon. The gall stone measuring 15 × 15 mm was removed, and it became evident that there was a fistula between the gall bladder and right transverse colon [Figure 3].

A cholecystectomy was performed with excision of the fistula and primary repair of the colon enterotomy. This was done in two layers after excising the cuff of the gall bladder that was initially kept on the wall of the colon to prevent creating a fistula. The patient made an uneventful recovery and was discharged on the 15th postoperative day. A histopathological examination of the specimen revealed no evidence of malignancy. Twelve weeks later, the CBD stent was removed after confirming a normal abdominal ultrasonography and ensuring that the liver function tests were within normal limits.

## DISCUSSION

Spontaneous cholecystocolic fistulae comprise 10–20% of all biliary-enteric fistulas. In the majority of cases, they are a sequel to cholecystitis but are reported to complicate only 0.13% cases.[2] However, they have also been reported in Crohn's disease, ulcerative colitis, abdominal trauma, and malignancy of the biliary tract, the bowel, and the head of the pancreas.[3]

Cholecystocolic fistulae can present with abdominal pain, nausea, weight loss, diarrhea, and dyspeptic symptoms. This fistula can alter the normal enterohepatic circulation of bile acids, leading to their malabsorption. The bile acids also stimulate the colonic mucosa directly to secrete water and electrolytes excessively, leading to steatorrhea and diarrhea.[4] Rare cases have been reported with the stone being impacted at the rectosigmoid causing large bowel obstruction due to such fistulae.[5]

If found incidentally during laparotomy, these fistulae should be taken down because of the increased risk of cholecystitis, cholangitis, and a 15% chance of malignancy in the gall bladder.[6]

The most useful techniques for diagnosis are a plain film of the abdomen, abdominal ultrasonography, barium studies, biliary scintigraphy, and ERCP. Although a diagnosis of cholecystocolic fistula is rarely suspected clinically, it should be considered in elderly patients with unexplained pneumobilia or unexplained persistent diarrhea. ERCP can be helpful in establishing the diagnosis, especially if barium studies give negative results.[7]

The standard treatment of a cholecystocolic fistula is open cholecystectomy and closure of the fistula. However, recent developments in laparoscopic surgery have shown its potential use in treating these rare fistulas. Similar techniques of open surgery have been used in laparoscopic surgery. The results have shown no significant differences in intraoperative and postoperative complications.[8] Although surgery is the treatment of choice, endoscopic sphincterotomy and CBD stone extraction have been said to cause spontaneous healing of the fistula by reducing increased biliary pressure. This has been used in the treatment of elderly unfit patients.[9]
